# Effects of spore powder of ganoderma lucidum on CaSR and apoptosis-related proteins in hippocampus tissue of epilepsy following dementia: A protocol of systematic review

**DOI:** 10.1097/MD.0000000000021711

**Published:** 2020-08-14

**Authors:** Li-hong Qin, Chen Wang, Xiao-xue Jiang, You Song, Yao Feng, Li-wei Qin, Shu-ping Zhang

**Affiliations:** a First Ward of Neurology Department; b Second Ward of Neurology Department; c Department of Quality Control Center; d Department of Chinese Medicine; e Department of Physical Diagnosis, First Affiliated Hospital of Jiamusi University, Jiamusi, China.

**Keywords:** dementia, epilepsy, spore powder of ganoderma lucidum

## Abstract

**Background:**

This study will investigate the effects of Spore Powder of Ganoderma Lucidum (SPGL) on CaSR and apoptosis-related proteins (ARP) in hippocampus tissue of epilepsy following dementia.

**Methods:**

This study will retrieve all potential studies from both electronic databases (Cochrane Library, EMBASE, MEDLINE, CINAHL, AMED, and CNKI) and other literature sources to assess the effects of SPGL on CaSR and ARP in hippocampus tissue of epilepsy following dementia. We will search all literature sources from the inception to the present. All eligible case-control studies will be included in this study. Two authors will independently carry out literature selection, data collection, and study quality evaluation. Any divergence will be resolved by another author through discussion. RevMan 5.3 software will be employed for data analysis.

**Results:**

This study will summarize existing evidence to assess the effects of SPGL on CaSR and ARP in hippocampus tissue of epilepsy following dementia.

**Conclusions:**

The findings of this study may provide helpful evidence of SPGL on CaSR and ARP in hippocampus tissue of epilepsy following dementia.

**Systematic review registration:**

INPLASY202070041.

## 1 Introduction

Epilepsy is a very common disorder, affecting about 65 million populations around the world.^[1–4]^ Its incidence is higher in the elderly people, especially for those patients over 65 years old, expect with almost 1 billion patients by 2030.^[5–7]^ It is more common in patients with dementia than general population.^[8,9]^ Although it is reported to be associated with alterations in inhibitory-excitatory systems, its mechanism is still not fully understood.^[10–12]^

There exists no effective treatment for both epilepsy and dementia. Fortunately, Spore Powder of Ganoderma Lucidum (SPGL) has reported to manage both of them.^[13–15]^ It is reported by exploring CaSR and apoptosis-related proteins (ARP) in hippocampus tissue of rats.^[16–32]^ However, no systematic review has been conducted to address this issue. Thus, this systematic review will investigate the effects of SPGL on CaSR and ARP in hippocampus tissue of rats with epilepsy after dementia.

## 2 Methods

### 2.1 Objective

This systematic review aims to evaluate the effects of SPGL on CaSR and ARP in hippocampus tissue of epilepsy following dementia.

### 2.2 Study registration

This study protocol has registered on INPLASY202070041. It has organized based on the standards of the Preferred Reporting Items for Systematic Review and Meta-Analysis Protocols Statement.^[33]^

### 2.3 Inclusion criteria for study selection

#### 2.3.1 Types of studies

All eligible case-control studies (CCSs) on assessing the effects of SPGL on CaSR and ARP in hippocampus tissue of rats with epilepsy following dementia will be considered for inclusion.

#### 2.3.2 Types of participants

Rats confirmed with epilepsy after dementia will be included in this study.

#### 2.3.3 Types of interventions

In the experimental group, any forms of SPGL were used as the only treatment.

In the control group, no restrictions were applied to the comparator, except SPGL.

#### 2.3.4 Types of outcome measurements

Outcomes include protein and gene expressions of CaSR, c-Fos, Caspase-3, Bcl-2, Bax, Neural Cell Adhesion Molecule 1, proliferating cell nuclear antigen, CyclinD1, livin; and levels of NO, NOS, and interleukin 10 in hippocampus tissue of rats.

### 2.4 Search methods for study identification

#### 2.4.1 Electronic bibliographic databases

We will search animal studies of question for SPGL on CaSR and ARP in hippocampus tissue of rats with epilepsy following dementia in Cochrane Library, EMBASE, MEDLINE, CINAHL, AMED, and CNKI from inception to the present. We present search strategy with details for Cochrane Library in Table 1. We will adapt similar search strategy to other electronic bibliographic databases.

**Table 1:**
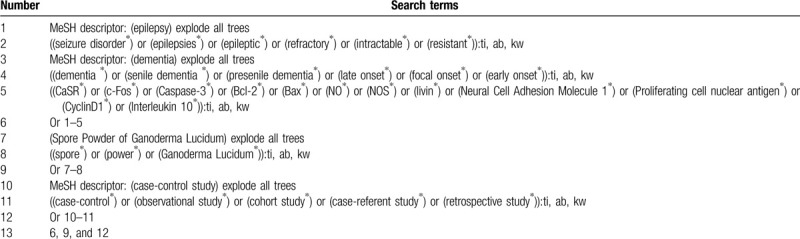
Search strategy of Cochrane Library Database.

#### 2.4.2 Other sources

We will also search other sources to avoid missing potential studies, such as conference proceedings, associated references lists of included studies, and ongoing trials from websites of clinical trial registry.

### 2.5 Data collection and analysis

#### 2.5.1 Study selection

Two authors will independently perform study selection according to the predefined eligibility criteria. If there are disagreements between both of them, we will invite another author to solve them. There are 2 stages in the process of study selection. At the first stage, we will scan titles/abstracts of all studies to remove duplicates and all irrelevant records. At the second stage, we will read full text of all potential articles to judge whether they can be finally selected in this study. The whole process of study selection will be presented in a flow diagram.

#### 2.5.2 Data extraction and management

Two authors will independently extract data from eligible study in accordance with a predefined standardized data extraction sheet. If we identify any disagreement, we will invite another author to solve it through discussion. We will extract the following information of title, first author, year of publication, species, gender, study methods, details of intervention and control (e.g. time, dosage, and duration), outcome indicators, results, and findings.

#### 2.5.3 Risk of bias assessment

The risk of bias of eligible CCSs will be performed by 2 independent authors using Newcastle-Ottawa Scale.^[34]^ If there is conflict between 2 authors, we will invite another author to clear up such division.

#### 2.5.4 Measurement of treatment effect

Treatment effect of outcome indicators will be estimated using risk ratio and 95% confidence intervals (CIs) for dichotomous data, and mean difference (MD) or standardized MD and 95% CIs for continuous data.

#### 2.5.5 Dealing with missing data

If any unclear or missing data occurs, we will contact primary author to obtain those data by email or phone. If those data is not achievable, we will analyze available data only, and will discuss its potential impacts.

#### 2.5.6 Data synthesis

We will use RevMan 5.3 software to analyze and synthesize outcome data. We will examine heterogeneity across CCSs using Cochrane *I*^*2*^ test. If the value of *I*^*2*^ ≤50% is identified, it means reasonable heterogeneity, and we will place a fixed-effects model. Under such situation, we will carry out meta-analysis if sufficient data is collected with sufficient similarity on the same outcome indicator. If the value of *I*^*2*^ > 50% is found, it indicates substantial heterogeneity, and we will apply a random-effects model. We will identify any possible sources of significant heterogeneity. If meta-analysis is deemed not to be performed, we will report study results by a narrative description.

#### 2.5.7 Reporting bias

We will check reporting bias using Funnel plot and Egger linear regression test to examine funnel plot asymmetry.^[35,36]^

#### 2.5.8 Subgroup analysis

We will undertake a subgroup analysis based on the different characteristics of subjects, treatment schedules, and outcome variables.

#### 2.5.9 Sensitivity analysis

We will carry out a sensitivity analysis to check the stability and robustness of study results according to the methodological quality, sample size, and missing data.

#### 2.5.10 Dissemination and ethics

We expect to publish this study through a peer-reviewed journal. This study does not need ethic approval, because we will only collect data from eligible studies.

## 3 Discussion

Although a variety of studies have reported the effects of SPGL on epilepsy and dementia,^[13–32]^ no systematic review investigated the effects of SPGL on CaSR and ARP in hippocampus tissue of rats with epilepsy after dementia. This systematic review will first summarize current available literature to assess the effects of SPGL on CaSR and ARP in hippocampus tissue of rats with epilepsy following dementia. We will include all potential eligible studies. The findings of this study will provide evidence to help judge whether SPGL is effective on CaSR and ARP in hippocampus tissue of rats with epilepsy after dementia.

## Author contributions

**Conceptualization:** Li-hong Qin, Chen Wang, Yao Feng, Shu-ping Zhang.

**Data curation:** Li-hong Qin, Xiao-xue Jiang, You Song.

**Formal analysis:** Xiao-xue Jiang, Yao Feng, Li-wei Qin.

**Funding acquisition:** Shu-ping Zhang.

**Investigation:** Shu-ping Zhang.

**Methodology:** Li-hong Qin, Chen Wang, Xiao-xue Jiang, You Song, Li-wei Qin.

**Project administration:** Shu-ping Zhang.

**Resources:** Li-hong Qin, Chen Wang, Xiao-xue Jiang, You Song, Yao Feng, Li-wei Qin.

**Software:** Li-hong Qin, Chen Wang, Xiao-xue Jiang, You Song, Yao Feng, Li-wei Qin.

**Supervision:** Shu-ping Zhang.

**Validation:** Li-hong Qin, Chen Wang, Xiao-xue Jiang, You Song, Li-wei Qin, Shu-ping Zhang.

**Visualization:** Li-hong Qin, Yao Feng, Li-wei Qin, Shu-ping Zhang.

**Writing – original draft:** Li-hong Qin, Chen Wang, Xiao-xue Jiang, You Song, Yao Feng, Shu-ping Zhang.

**Writing – review & editing:** Li-hong Qin, Chen Wang, Yao Feng, Li-wei Qin, Shu-ping Zhang.
